# The Impact of Green Innovation on Enterprise Green Economic Efficiency

**DOI:** 10.3390/ijerph192416464

**Published:** 2022-12-08

**Authors:** Yuan Li, Nan Huang, Yang Zhao

**Affiliations:** 1 School of Management, Jinan University, Guangzhou 510632, China; 2 Research Center of Low Carbon Economy for Guangzhou Region, Jinan University, Guangzhou 510632, China; 3 School of Accounting, Nanjing Audit University, Nanjing 211815, China

**Keywords:** green innovation, green economic efficiency, high-quality development

## Abstract

In the process of China’s transformation from high-speed to high-quality development, the role of green innovation has gradually begun to receive attention. Using 2163 observations of 687 listed companies from 2016 to 2020, this paper examined whether green innovation can improve green economic efficiency. The study found that green innovation significantly reduces the green economic efficiency of enterprises. In the case of insufficient protection of innovation achievements, limited knowledge and technology accumulation, and the absence of enterprise engagement in heavily polluting production and operation activities, the negative impact of green innovation on their green economic efficiency is more significant. Moreover, upgraded production processes, a high degree of freedom of technology selection and high market competition can help alleviate the negative impact of green innovation on enterprises’ green economic efficiency, and when the above three conditions are simultaneously met, green innovation significantly promotes the green economic efficiency of enterprises. The above findings are contradictory to the assumption of most literature intuitively. However, after a series of tests, this paper found that green innovation can still stimulate overall environmental and economic performance in some conditions. Starting from the microenterprise level and based on actual emissions data, this paper examines whether and how green innovation affects high-quality development. The findings are of great significance to academic research, policy formulation, and enterprise production and operation.

## 1. Introduction

With economic growth and environmental problems becoming increasingly serious, the need for China to change its development model is growing. The report of the “19th National Congress of the Communist Party of China” pointed out that China’s economic growth model has begun to transform from high speed to high quality. In this transformation process, technological innovation plays an important role. The Sixth Plenary Session of the 19th CPC Central Committee further communicated that the transformation of a high-quality, efficient and sustainable economic growth model cannot be separated from the support of scientific and technological strength. It can be seen that China’s requirements have gradually changed from needing high-speed development to needing high-quality development as its economic status has improved. Moreover, innovation has played an important role in the process of changing China’s development mode. Therefore, investigating whether and how innovation activities can meet the requirements for high-quality development has important theoretical and practical significance.

Green innovation, also known as green technology innovation, refers to technological innovation that takes into account environmental factors and usually has the dual attributes of economic development and energy conservation with emission reduction. In contrast to traditional innovation, green innovation also gives substantial consideration to reducing environmental pollution while developing the economy. Because green innovation can not only reduce pollution and energy consumption but also bring economic benefits to enterprises, it plays a key role in the high-quality transformation of economic development. However, innovative behaviours may have a negative impact on firm financial performance due to inherent risk-benefit issues [1]. In addition, green innovation is more difficult and riskier than traditional innovation, and its risk-benefit problem is more serious than that of traditional innovation [2]. To test whether green innovation can affect overall environmental and economic performance, this paper starts from the perspective of green economic efficiency and provides empirical evidence that green innovation affects the high-quality development of enterprises.

Improvements in green economic efficiency mean increases in the overall performance of the environment and economy, which are direct manifestations of high-quality development. Green economic efficiency incorporates both environmental and economic factors into the analytical framework to avoid biases in performance evaluations caused by one-sided attention given to one factor, so it is widely used in the evaluation of overall environmental and economic performance [3,4,5,6]. Green innovation is an innovative activity that takes into account both economic development and energy conservation and emission reduction. Testing the effects of green innovation on overall environmental and economic performance can enable a more accurate evaluation of its economic consequences to better understand whether and how it can affect high-quality development.

This paper uses as a sample A-share listed companies in Shanghai and Shenzhen from 2016 to 2020 to test the impact of green innovation on enterprises’ green economic efficiency and identify how green innovation affects their overall environmental and economic performance and high-quality development. The study finds that green innovation reduces the green economic efficiency of enterprises, and the above conclusion still holds after the robustness test. Further analysis shows that upgraded production processes, a higher degree of freedom of technological selection, and higher market competition intensity can help alleviate the negative effects of green innovation on the green economic efficiency of enterprises. In addition, adequate protection of innovation achievements and the high-level accumulation of knowledge and technology can reduce the negative impact of green innovation on enterprises’ green economic efficiency, and the characteristics of green innovation that are related to environmental performance make such innovation more profitable for heavily polluting enterprises. The realization of the innovation compensation enables green innovation to have a positive impact on the green economic efficiency of enterprises when the three conditions of production process upgrades, a higher degree of freedom in technology selection, and higher market competition intensity are satisfied at the same time. The above findings show that supporting conditions are needed to promote high-quality development with green innovation, and the relevant systems should be improved to promote green innovation in an orderly manner rather than blindly playing a role in promoting high-quality development.

The main contributions of this paper are as follows. First, this paper examines the effect of green innovation on overall environmental and economic performance and provides empirical evidence on whether and how green innovation affects high-quality development. Most of the literature assumes that green innovation has positive economic consequences and notes that an increase in green innovation means improvements in development quality, and discussions are based on these findings [7,8,9,10]. By examining the impact of green innovation on the green economic efficiency of enterprises, this paper verifies the actual effect of green innovation on overall environmental and economic performance and finds that a high level of green innovation and high-quality development are not identical. This finding deepens the understanding of the economic consequences of green innovation by providing empirical evidence of green innovation’s effect on the green economic efficiency of enterprises.

Second, this paper is the first to start from the microenterprise level and use actual emissions data to test the impact of green innovation on green economic efficiency. The literature discussion on whether green innovation can improve the green economic efficiency is limited, and their researches have deficiencies. For instance, they failed to use enterprise-level emissions data [11] or use enterprise-level emissions data of questionable reliability [12]. This paper uses actual emissions data to calculate the green economic efficiency of enterprises and tests the effect of green innovation on their overall environmental and economic performance based on this to verify whether and how green innovation can affect high-quality development. By providing more valuable and reliable empirical evidence, this paper indicates whether and how green innovation can affect overall environmental and economic performance.

Third, based on a classical theory, this paper investigated why the green innovation has a negative impact on the green economic efficiency of enterprises, and how to reverse the impact to a positive. Intuitively, green innovation can improve overall environmental and economic performance, yet empirical evidence based on microenterprise level and actual emissions data does not support the above view. To explore the causes, this paper tests the moderating effects of external and internal environments heterogeneity. Moreover, based on a classical framework which indicates conditions of realization of innovation compensation [13,14], this paper provides a possible solution to adverse the negative impact of green innovation on the green economic efficiency of enterprises. The above findings are significant to policy formulation and enterprise production and operation.

Section 2 provides the literature analysis and hypothesis. Section 3 describes the Methodology. Section 4 sheds light on the results of the study. Finally, Section 5 highlights the conclusions, recommendations, and limitations.

## 2. Literature Analysis and Hypothesis

### 2.1. Literature Analysis

Compared with traditional innovation, addressing environmental performance is the main highlight of green innovation. Green innovation has significantly reduced pollution and carbon emissions by reducing consumption, providing end-of-pipe treatments, and improving resource utilization, which has positive impacts on environmental performance [8,9,10]. Green innovation can also stimulate the role of intellectual capital and human resource management and promote the green transformation of enterprises, thereby reducing resource consumption and pollution levels [15]. In addition, green innovation can improve corporate environmental awareness, thereby promoting corporate environmental performance [8,16]. Due to the rise in environmental performance, evaluations of companies by stakeholders have improved [17,18,19], the corporate image has been improved [20,21], and Environment, Social, and Governance (ESG) performance has been significantly improved [22].

However, the impact of green innovation on economic performance is inconclusive. Some researchers consider that although higher investment is needed, the innovation on economic performance can still be positive. For instance, Porter’s hypothesis, proposed by Michael Porter, maintains that innovation triggered by environmental regulations can bring compensation effect to enterprise [13,14]. By reducing energy consumption, green innovation reduces production costs for enterprises, which helps directly improve their profitability [23], relax the financing constraints they face [19], reduce their financial risks [24], and improve their value [25,26], market competitiveness and returns on investment [9,20]. In addition to directly improving the economic performance of enterprises, green innovation can have an indirect impact on economic performance, such as enhancing the corporate image to generate price premiums [27], attracting investment and subsidies to ease financing constraints [18], reducing pollution to improve reputations and increase market share [8], managing environmental risks, fulfilling social responsibilities and supplementing organizational learning abilities to strengthen competitive advantages [17,28]. In addition, the high demand for capital for green innovation drives provincial financial development [29], and the additional opportunities it brings have increased employment rates [30]. Therefore, much of the literature has regarded the rising level of green innovation as a sign of improved development quality, has assumed that green innovation will have positive economic consequences, and has conducted research on this basis [7,31,32,33].

On the other hand, since innovation, especially green innovation, is a high-investment and high-risk production and operation behaviour, the cost of green innovation for enterprises cannot be ignored, and in many cases, its benefits cannot offset the costs [34]. Therefore, the Porter hypothesis has been questioned since its conception [35]. Due to the higher cost of green innovation, it results in more serious risk–benefit problems than traditional innovation [36], which may lead to negative economic consequences, such as declining financial performance, lower productivity, and pessimistic analyst forecasts [1,35,37,38,39].

Because the characteristics of green innovation affect both environmental performance and economic performance, some studies have attempted to explore the impact of green innovation on the overall performance of the environmental economy to conduct a more comprehensive evaluation of its economic consequences. At the macro level, some studies have examined the impact of green innovation on the overall performance of the provincial environment and economy and have found that green innovation improves green total factor productivity by promoting provincial energy conservation, emissions reductions, and technological progress, and the above effects are stronger in eastern regions, northern provinces and regions with moderate pollution [11]. At the enterprise level, studies have used regional industrial output value, enterprise income, and total regional emissions to estimate the emissions levels of enterprises, have used this estimate to test the effect of green innovation on enterprises’ overall environmental and economic performance, and have found that green innovation plays a greater role in improving green total factor productivity and that its promotional effect on green total factor productivity is more significant in state-owned and heavily polluting enterprises [12].

In summary, existing studies have included in-depth discussions of green innovation but still have deficiencies. First, existing research has been based on the assumption that green innovation has positive economic consequences and has subjectively regarded it as a sign of high-quality development, ignoring the uncertainty of the actual economic consequences. Second, most of the existing studies have focused on the effect of green innovation on environmental or economic performance and have failed to test its effect on overall environmental and economic performance. Although some studies have examined the effect of green innovation on overall environmental and economic performance, they may have failed to start at the enterprise level or the reliability of the data used was deficient [11,12]. Therefore, this paper starts from the enterprise—the main body engaged in green innovation and other production and operation activities—and uses actual enterprise pollution emissions data and green economic efficiency to examine the effect of green innovation on overall environmental and economic performance and high-quality development.

### 2.2. Theoretical Analysis and Hypothesis Development

Innovation theory holds that changes in the production and business environment induce enterprise innovation activities [40]. With the implementation of environmental regulations, corrections to external diseconomies internalize environmental problems, and environmental problems cause social costs to be borne by enterprises [41]. At this time, enterprises as “economic entities” change their production and operation behaviours, such as carrying out innovative activities that take into account environmental performance, to avoid punishment [30]. Needing to deal with environmental regulations is also an important motivation for enterprises to carry out green innovation [42]. Green innovation increases enterprises’ costs, resulting in damage to their economic interests [37]. However, if the innovation compensation can achieve, the subsequent improvement in their technological levels can enable them to achieve a win–win” for environmental and economic performance [13,14].

Green economic efficiency reflects the overall environmental and economic performance. Theoretically, decreasing input such as capital and labor, increasing desired output such as economic benefits, and decreasing undesired output such as emissions can all help to improve the green economic efficiency of enterprises [43]. In contrast to traditional innovation, green innovation focuses on environmental performance, such as energy conservation and emissions reductions, and may create a series of economic benefits due to improvements in environmental performance [13,14]. First, green innovation helps save energy and reduce consumption, which can reduce enterprises’ production costs and directly provide them with economic benefits [9,19,20,23,24,25,26]. Second, by reducing pollution emissions, green innovation can indirectly improve the economic performance of enterprises. Reductions in pollution emissions-free up companies from dealing with regulatory penalties, enabling green innovation to protect companies’ economic benefits in a disguised form [9]. Because of the focus on environmental performance, the corporate image is improved [27], reputations are improved [8], stakeholder evaluations become more positive [18,19], and environmental risk management capabilities are enhanced [17]. This helps enterprises attract investments and subsidies to ease their financing constraints and improves their value, market competitiveness, and market share [8,18,25]. Regarding simultaneously improving environmental and economic performance, green innovation will have a positive impact on the overall environmental and economic performance of enterprises.

However, if the Porter hypothesis is established, in the case of the internalization of environmental costs, corporate innovation behaviour should increase significantly; however, this is not the case [44]. One possible reason is that the application of new technologies can indeed bring certain benefits to enterprises, but it is difficult to make up for the costs [35], so whether innovation compensation can achieve a “win-win” situation is still worth exploring. The literature also pointed out that the realization of innovation compensation is inseparable from the following conditions [14]: First, the realization of innovative compensation requires production processes to be upgraded rather than engaging purely in pollution control. The production process upgrade itself is very difficult to realize, and the probability of failure is high. The constraints of green innovation when taking into account environmental performance make the above situation even worse, and green upgrades of production processes increase an enterprise’s economic burden [36]. If it is difficult to upgrade the production process, the green innovation of pure pollution control has become the choice of enterprises. At this time, green innovation increases an enterprise’s costs and does not easily bring economic benefits, adversely affecting its green economic efficiency. Second, to achieve innovation compensation, companies need to freely choose the technologies to be applied. The consideration of environmental performance with respect to green innovation prevents companies from purely benefitting economically when carrying out innovation activities [25], limiting their optional technologies and preventing them from choosing the most economical solution according to their own conditions. As a result, the enterprise’s economic benefits are reduced and its green economic efficiency is adversely affected. Third, sensitive costs and positive market incentives are also important conditions for the realization of innovation compensation [13]. Green innovation is a high-cost production and operation behaviour [1]. It increases enterprises’ costs and does not easily bring economic benefits, which leads to a decline in their green economic efficiency. In summary, the consideration of environmental performance with respect to green innovation increases enterprises’ economic burden, restricts their pursuit of optional technologies and thus has an adverse impact on their green economic efficiency. Moreover, if external and internal environments for enterprise production and operation prevent an enterprise from obtaining enough innovation benefits, the adverse impact of green innovation may be further increased. Accordingly, this paper proposes the following hypothesis.

**Hypothesis** **1.** *Green innovation reduces corporate green economic efficiency.*

**Figure 1:**
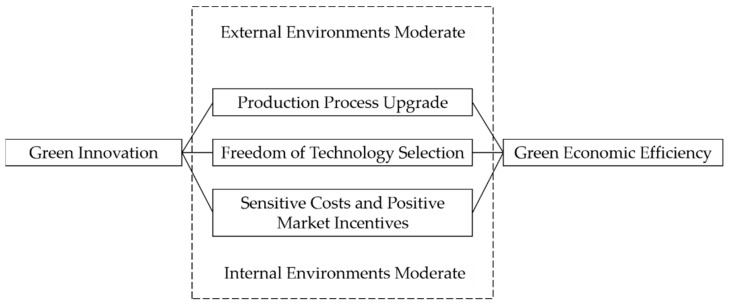
Theoretical logic diagram.

Based on the above discussions, a theoretical logic diagram is developed, which is shown in Figure 1.

## 3. Methodology

### 3.1. Samples and Data

According to the “Standards for the Contents and Formats of Information Disclosure by Companies Offering Securities to the Public No. 2”, revised by the China Securities Regulatory Commission in 2016, if the company or its important subsidiaries are key pollutant dischargers as announced by the Ministry of Ecology and Environment of the People’s Republic of China, the information on the pollutant discharges should be disclosed. Therefore, this paper selects listed companies from 2016 to 2020 as the research sample and processes them as follows: samples with missing data and ST companies are deleted, and all continuous variables are Winsorized by 1% to eliminate the influence of outliers data. After the above processing, this paper obtained 2163 observations of 687 listed companies for the research. The corporate pollution emissions data used in this study are collected from the annual reports of listed companies, the green innovation data are from the Chinese Research Data Services database (CNRDS), and the rest of the data are from the China Stock Market and Accounting Research database (CSMAR).

### 3.2. Variable Definition and Research Model

#### 3.2.1. Green Innovation

It takes a certain amount of time from patent application to acquisition, so this paper uses the ratio of the number of green patent applications of enterprises to the total number of patent applications to measure the level of green innovation of enterprises.

#### 3.2.2. Green Economic Efficiency

This paper uses the nonradial directional distance function (Non–radial DDF) proposed by Zhou et al. (2012) to measure the green economic efficiency of enterprises [3]. First, this paper defines environmental production technology as in model (1), where *k* is the capital input, *l* is the labour input, *y* is the desired output, *b* is the undesired output, and *P* is the production possibility set:

(1) P={(k,l,y,b):(k,l) can produce (y,b)}

Subsequently, this paper defines the Nonradial DDF as shown in model (2):

(2) $$\vec{ND}(k,l,y,b;g)=\sup\{w^{T}\beta :[(k,l,y,b)+g\times diag(\beta )]\in P\}$$

In model (2), w is the weight vector, g is the directional vector, and *β* ≥ 0 is the vector of the scaling factors. For research needs, this paper sets the directional vector *g = (−k, −l, y, −b)* and assigns the capital and labour inputs each a weight of 1/6, assigns the expected and undesired outputs each a weight of 1/3, and sets the weight vector to *w* = (1/6, 1/6, 1/3, 1/3). To obtain the optimal solution, this paper solves the DEA model shown in model (3):

(3) $$\begin{array}{l} \vec{ND}(k,l,y,b;g)=\max w_{k}\beta_{k}+w_{l}\beta_{l}+w_{y}\beta_{y}+w_{b}\beta_{b}\\ s.t.\sum_{n=1}^{N}\theta_{n}k_{n}\leq k-\beta_{k}g_{k}\\ \;\;\;\;\sum_{n=1}^{N}\theta_{n}l_{n}\leq l-\beta_{l}g_{l}\\ \;\;\;\;\sum_{n=1}^{N}\theta_{n}y_{n}\geq y+\beta_{y}g_{y}\\ \;\;\;\;\sum_{n=1}^{N}\theta_{n}b_{n}=b-\beta_{b}g_{b}\\ \;\;\;\;\theta_{n}\geq 0,n=1,\cdots ,N\\ \;\;\;\;\beta_{k},\beta_{l},\beta_{y},\beta_{b}\geq 0 \end{array}$$

Solving model (3) can obtain the optimal solution $\beta_{k}^{*}$, $\beta_{l}^{*}$, $\beta_{y,}^{*}$ and $\beta_{b}^{*}$, which represents capital input inefficiency, labor input inefficiency, desired output inefficiency, and undesired output inefficiency respectively. Referring to the practice of Zhang et al. (2014) [43], this paper defines green economic efficiency (GEE) as the average efficiency of each factor, which can be formulated as modeled (4):

(4) $$GEE=\frac{1-1/3\times (\beta_{k}^{*}+\beta_{l}^{*}+\beta_{b}^{*})}{1+\beta_{y}^{*}}$$

The *GEE* lies between zero and unity. The larger the *GEE* is, the higher the green economic efficiency of an enterprise and the better its overall environmental and economic performance. *GEE* equals unity means the enterprise has the best overall environmental and economic performance. Based on model (4), it is obvious that both increase in capital input, labor input, or undesired output and a decrease in desired output will aggravate inefficiency, and make the green economic efficiency lower.

This paper uses the following input-output variables: the capital input variable represents total fixed assets; the labour input variable represents the number of employees; the desired output variable represents operating income; the undesired output variable represents the “environmental tax” calculated based on the company’s sulphur dioxide, nitrogen oxide, and chemical oxygen demand, ammonia nitrogen emissions and the minimum standards of the “Environmental Protection Tax Law of the People’s Republic of China”. At the same time, this paper deflates variables related to price.

#### 3.2.3. Control Variables

In line with the research questions, this paper selects enterprise size (Size), return on assets (ROA), the asset-liability ratio (Lev), accounts payable turnover ratio (APTR), growth (Growth), price-earnings ratio (PER), management fees rate (MF), risk-taking level (RT), ownership concentration (Share), the proportion of independent directors (Inde), two-in-one (CEO), and enterprise age (Age) as control variables. In addition, this paper controls the year and firm fixed effect; see Table A1 for details.

In summary, this paper builds model (5) to test hypothesis 1 in this paper:

(5) $$GEE_{i,t}=\alpha_{0}+\alpha_{1}GI_{i,t}+\alpha_{2}Controls_{i,t}+\lambda_{t}+\nu_{i}+\varepsilon_{i,t}$$

In model (5), *GEE_i,t_* denotes the green economic efficiency of firm *i* in year *t*. *GI_i,t_* denotes the green innovation level of firm *i* in year *t*. *Controls_i,t_* denotes the control variables of firm *i* in year *t*. *λ_t_* denotes the year-fixed effect. *ν_i_* denotes the firm fixed effect. *ε_i,t_* denotes the error term. In this paper, model (5) is used for the regression analysis using the whole sample. If *α*_1_ is statistically significantly negative, then H1 is confirmed; in contrast, if α_1_ is not significant or is significantly positive, then H1 does not hold. At the same time, considering that the disturbance items at the enterprise level may be correlated, the regression analysis in this paper uses the cluster of robust standard errors at the enterprise level.

## 4. Results

### 4.1. Descriptive Statistics

Table 1 reports the descriptive statistics results, which show that the mean of GEE is 0.0840, the median is 0.0546, and the maximum and minimum values are 0.0007 and 0.6145, respectively; the mean of GI is 0.1238, the median is 0.0606, and the maximum and minimum values are 0.0000 and 0.8462, respectively. This result shows that the sample enterprises have certain differences in green economic efficiency and green innovation. In addition, Table 1 lists the descriptive statistical results of the other variables. The mean, median and extreme values show certain differences in the characteristics of the sample enterprises. However, the effects of extreme values were removed after Winsorizing the continuous variables. A brief explanation of Winsorizing has been provided in Appendix A. Moreover, the descriptive statistics results of the input and output of the DEA model are shown in Table A2.

### 4.2. Green Innovation and Green Economic Efficiency

Table 2 reports the test results of Hypothesis 1. Columns (1)–(4) of Table 2 show the results when no control variables are added, financial control variables are added, governance control variables are added, and all control variables are added respectively. All absolute values of the coefficients of GI are greater than 0.0150 and statistically significantly negative at the 5% level, indicating that for every unit increase in the level of green innovation, the green economic efficiency will statistically significantly reduce over 0.0150 units. Moreover, based on coefficients and standard deviation of GI, and mean of GEE, one standard deviation increase in the level of green innovation will lead to over 3.0285% decrease in green economic efficiency relative to the mean. The above results indicate that green innovation has a significant negative effect on the green economic efficiency of enterprises both statistically and economically. Hypothesis 1 is proved by this finding.

### 4.3. Robustness Test

#### 4.3.1. Sample Selection Bias

As mentioned above, due to the low green innovation of the sample companies, if there is sample selection bias, the reliability of the regression results is affected. Therefore, to avoid the influence of sample selection bias on the regression results, this paper uses the propensity score matching method to rematch the research samples and the regression analysis is conducted. Specifically, this paper divides the green innovation level of enterprises into two groups based on the mean value and uses the ratios of 1:1 and 1:4. Size, ROA, Lev, APTR, Growth, PER, MF, TobinQ, Share, Inde, CEO and Age are selected variables for matching with replacement to finally obtain 1238 and 1872 observations, respectively. Table A4 and Table A5 list the matched results. The results of the *t*-test show that after matching, the difference between the original samples is greatly reduced, the difference is no longer significant, and the screening effect is eliminated. Table A6 and Table A7 report the ATT value during data matching. The results of the *t*-test show that the average treatment effect on the treated is statistically significant, indicating that green innovation has a negative impact on the green economic efficiency of enterprises.

The above samples are substituted into model (5) for the regression, and the results are shown in Table 3. Table 3 shows that whether the samples are matched in 1:1 or 1:4 ratios and whether or not control variables are added, the GI coefficient is statistically significantly negative, and the absolute value of the coefficient has increased, which is statistically and economically consistent with the previous regression analysis results. The conclusion of this paper is robust.

#### 4.3.2. Omitted Variable Problem

There may be unobservable factors that affect the green economic efficiency of enterprises, leading to the problem of omitted variables and, thus, influencing the robustness of the conclusions of this paper. To solve the problem of omitted variables, this paper uses the industry-regional average level of green innovation (IV) as an instrumental variable to perform 2SLS regression. Theoretically, triggered by excepted competitive advantages [12,13], the average level of industry-regional green innovation affects the green innovation decision-making of enterprises. However, the green economic efficiency of an enterprise depends on its own production technology rather than others, the average level of industry-regional green innovation has no direct impact on their green economic efficiency. The results of the correlation analysis are shown in Table A9, it is obvious that the relationship between the industry-regional average level of green innovation and the green economic efficiency of enterprises is statistically insignificant.

The 2SLS regression analysis results are shown in columns (1)–(2) of Table 4. The coefficient of IV is 0.9797 and is statistically significant at the 1% level. The coefficient of GI is −0.0163 and is statistically significant at the 10% level, indicating that the average level of industry-regional green innovation significantly stimulates the green innovation behaviour of enterprises, and the green innovation behaviour of enterprises significantly reduces their green economic efficiency. In addition, the Kleibergen–Paap rk LM statistic was 59.759, which passes the underidentification test; the Cragg-Donald Wald F statistic was 1762.702, and the Kleibergen–Paap rk Wald F statistic was 1182.870, which passes the weak instrumental variable test and indicates that the instrumental variables are valid. In summary, after dealing with the problem of missing variables, green innovation still has a statistically and economically significant negative effect on the green economic efficiency of enterprises, and the conclusions of this paper are robust.

#### 4.3.3. Self-Selection Bias

The negative impact of green innovation on green economic efficiency has been demonstrated above, but there is still a factor that affects the robustness of the conclusions of this paper that needs to be excluded. That is, enterprises that carry out green innovation may have low green economic efficiency, which may lead to the existence of self-selection problems. To address this possible endogeneity problem, this paper uses the Heckman two-stage method. First, this paper selects the variables that affect the green innovation of enterprises—the industry-regional average level of green innovation (IV), Size, Size, ROA, Lev, APTR, Growth, PER, MF, RT, Share, Inde, CEO and Age—and performs probit regression to estimate the inverse Mill’s ratio (IMR). After that, the obtained IMR is substituted into model (5) for the regression, and the results are shown in columns (3)–(4) of Table 4. Column (4) of Table 4 shows that the coefficient of GI is −0.0213, still statistically significant at the 5% level, indicating that after controlling for the self-selection problem, the green innovation still has a statistically and economically significant negative impact on the green economic efficiency of enterprises, and the conclusion of this paper is robust.

### 4.4. Further Analysis

The regression analysis above verifies that green innovation has a significant negative impact on the green economic efficiency of enterprises. According to theoretical expectations, the realization of innovation compensation requires production process upgrades, free selection of available technologies, sensitive costs, and positive market incentives [13]; otherwise, innovation simply increases enterprises’ burdens. Accordingly, this paper further examines the impact of green innovation on the green economic efficiency of enterprises from the above perspectives to deepen the understanding of the relationship between the two.

#### 4.4.1. Production Process Upgrade

As mentioned above, it is more difficult to upgrade the production process, and the probability of upgrade failure is high [36], which increases the economic burden on enterprises and causes green innovation to adversely affect their green economic efficiency. However, if the production process is upgraded, the income generated improves the economic performance of the enterprise, thereby alleviating the negative effect of green innovation on its green economic efficiency. Generally, green invention innovation involves higher technical content, can easily create a competitive advantage to improve innovation compensation, and is generally considered to be representative of production process upgrades; green utility model innovations are easy to operate and are often used for end-of-pipe treatment [45]. To examine the impact of production process upgrades on the relationship between green innovation and enterprises’ green economic efficiency, this paper divides enterprise patents into green invention innovation and green utility model innovation for testing. Specifically, this paper uses the ratio of corporate green invention innovation to invention innovation to measure the level of corporate green invention innovation (GI-I) and the ratio of corporate green utility model innovation to utility model innovation to measure the level of corporate green utility model innovation (GI-UM). Regression analysis was performed by replacing the independent variables in the model (5) with the above variables. The results are shown in columns (1)–(2) of Table 5. The coefficient of GI-I is −0.0096, which is not statistically significant; the coefficient of GI-UM is −0.0142, which is statistically significant at the 5% level, indicating that for every unit increase of the level of green invention innovation, the green economic efficiency will statistically insignificantly reduce 0.0096 unit. Furthermore, for every unit increase in the level of green utility model innovation the green economic efficiency will statistically significantly reduce by 0.0142 units. Compared with green invention innovation, green utility model innovation has a more significant negative impact on green economic efficiency statistically and economically. This result shows that production process upgrades can help alleviate the negative effect of green innovation on the green economic efficiency of enterprises.

#### 4.4.2. Freedom of Technology Selection

The requirement for green innovation to take into account environmental performance limits the applicable technologies of enterprises, preventing them from carrying out innovation activities with the most economical solution and, thus, leading to a decline in enterprises’ green economic efficiency. However, if the technology supply conditions are good, the technology options that enterprises can choose increase, which helps improve their freedom of technology selection and, thereby, alleviates the adverse impact of green innovation on their green economic efficiency. Accordingly, this paper divides the sample into two groups, “high-tech market” and “low-tech market”, to enable the regression analysis to verify the above expectations. Specifically, referring to the standard of Wang et al. (2021) [46], if the marketization index of technological achievements in the region where the enterprise is located is higher than the median, it is classified as a “high-tech market”; otherwise, it is classified as a “low-tech market”. The above two groups of samples are substituted into model (5) for the regression analysis, and the results are shown in columns (3)–(4) of Table 5. The GI coefficient of the “high-tech market” group is −0.0154 and is not statistically significant; the coefficient of the “low-tech market” is −0.0199 and is statistically significant at the 5% level, indicating that when technology supply conditions are good, every unit increase in the level of green innovation will statistically insignificantly reduce the green economic efficiency for 0.0154 unit. Therefore, when technology supply conditions are bad, every unit increase in the level of green innovation will statistically significantly reduce the green economic efficiency for 0.0199 units. The better technology supply conditions are, the green innovation has the less negative impact on green economic efficiency. This shows that the improvement in the degree of freedom of technological selection can help alleviate the negative impact of green innovation on the green economic efficiency of enterprises.

#### 4.4.3. Intensity of Market Competition

The cost increase caused by green innovation reduces their economic benefits, thereby reducing their green economic efficiency. However, higher market competition makes costs more sensitive and brings positive incentives. Therefore, higher market competition intensity helps mitigate the adverse effects of green innovation on the green economic efficiency of enterprises [13]. Accordingly, this paper divides the samples into two groups, “high market competition” and “low market competition”, for the regression analysis to verify the above expectations. Specifically, this paper calculates the Herfindahl–Hirschman Index (HHI) based on total assets to measure the intensity of the market competition. If an enterprise’s HHI is below the median, it is classified as “high market competition”; otherwise, it is classified as “low market competition”. The above two groups of samples are substituted into model (5) for the regression analysis, and the results are shown in columns (5)–(6) of Table 5. The GI coefficient of the “high market competition” group is −0.0108 and is not statistically significant; the coefficient of “low market competition” is −0.0185 and is statistically significant at the 10% level, indicating that when market competition intensity is high, every unit increase of the level of green innovation will statistically insignificantly reduce the green economic efficiency for 0.0108 unit. Therefore, when market competition intensity is low, every unit increase in the level of green innovation will statistically significantly reduce the green economic efficiency for 0.0185 units. The higher the market competition intensity is, green innovation has the less negative impact on the green economic efficiency. This result shows that high market competition intensity can help alleviate the adverse effects of green innovation on the green economic efficiency of enterprises.

### 4.5. Extended Test

The above regression analysis results show that upgraded production processes, a high degree of freedom of technology selection, and high market competition intensity can help alleviate the negative impact of green innovation on the green economic efficiency of enterprises. In addition, the heterogeneity of external and internal environments for enterprise production and operation may moderate the relationship between green innovation and enterprises’ green economic efficiency. To explore the moderating effects of external and internal environments, this paper employs an extended test to investigate the influence of the degree of protection of innovation achievements and the nature of the production and operation activities of enterprises.

#### 4.5.1. The Degree of Protection of Innovation Achievements

Protecting innovation achievements from being imitated is an important condition for enterprises to obtain benefits from their innovative behaviour [47]. To maintain competitive advantages resulting from innovation, enterprises take actions to protect their innovation achievements, such as applying for patents [48]. Currently, the degree of protection of innovation achievements affects the amount of the benefits that enterprises can obtain from their innovative behaviours. In the case of a higher degree of protection of innovation achievements, innovation behaviours are more likely to bring competitive advantages to enterprises, enabling them to obtain higher innovation benefits and alleviate the negative effects of innovation behaviours. Regarding the research question in this paper, a high level of protection of innovation achievements can help improve the benefits that enterprises obtain from green innovation, which is likely to alleviate the negative impact of green innovation on the green economic efficiency of enterprises. To verify the above expectations, this paper divides the samples into the two groups of “strong legal environment” and “weak legal environment” and the two groups of “strong technical protection” and “weak technical protection” for the regression analysis. Specifically, based on the “Maintaining the Legal Environment of the Market” index and the “Intellectual Property Protection” index proposed by Wang et al. (2021) [46], if the “Maintaining the Legal Environment of the Market” index in the region where the business is located is higher than the median, the region is classified as a “strong legal environment”; otherwise, it is classified as a “weak legal environment”. If the “Intellectual Property Protection” index of the region where the enterprise is located is higher than the median, the region is classified as having “strong technical protection”; otherwise, it is classified as having “weak technical protection”. The above two groups of samples are substituted into model (5) for the regression analysis, and the results are shown in Table 6. Columns (1)–(2) of Table 6 show that the GI coefficient of the “strong legal environment” grouping is −0.0067, which is not statistically significant; the coefficient for the “weak legal environment” grouping is −0.0152 and statistically significant at the 10% level. Columns (3)–(4) of Table 6 show that the GI coefficient of the “strong technical protection” group is −0.0129, which is not statistically significant; the coefficient of “weak technical protection” is −0.0174 and statistically significant at the 10% level, indicating that when the legal environment (technical protection) is strong, every unit increase of the level of green innovation will statistically insignificantly reduce the green economic efficiency for 0.0067 (0.0129) unit. Therefore, when the legal environment (technical protection) is weak, every unit increase in the level of green innovation will statistically significantly reduce the green economic efficiency for 0.0152 (0.0174) units. The stronger the legal environment (technical protection) is, green innovation has the less negative impact on green economic efficiency. This result shows that with an improvement in the degree of protection of innovation achievements, the innovation benefits obtained by enterprises also increase, helping to alleviate the negative impact of green innovation on green economic efficiency.

#### 4.5.2. The Nature of the Production and Operation Activities of Enterprises

An increase in the level of knowledge and technology accumulated by an enterprise increases its innovation income [49], which may help alleviate the negative effect of its innovation behaviour on itself. Due to their high-intensity innovation activities, high-tech enterprises have accumulated more knowledge and technology than non-high-tech enterprises. This may help improve the innovation benefits obtained by enterprises, thereby mitigating the negative impact of green innovation on their green economic efficiency. To verify the above expectations, this paper divides the sample into two groups, “high-tech enterprises” and “non-high-tech enterprises”, for the regression analysis. Specifically, if an enterprise is engaged in a high-tech industry specified in the “Classification of High-Tech Industries (Manufacturing) (2017)” issued by the National Bureau of Statistics of China, it is classified as a “high-tech enterprise”; otherwise, it is classified as a “non-high-tech enterprise”. The above two groups of samples are substituted into model (5) for the regression analysis, and the results are shown in columns (1)–(2) of Table 7. The results show that the GI coefficient of the “high-tech enterprises” group is −0.0108 and not statistically significant; the coefficient of “non-high-tech enterprises” is −0.0171 and statistically significant at the 5% level, indicating that when an enterprise engages in high-tech production and operation activities, every unit increase of the level of green innovation will statistically insignificantly reduce the green economic efficiency for 0.0108 unit. Otherwise, every unit increase in the level of green innovation will statistically significantly reduce the green economic efficiency by 0.0171 units. This shows that high-tech enterprises can improve their innovation income by accumulating high-level knowledge and technology, thereby alleviating the negative impact of green innovation on their green economic efficiency.

In addition to economic factors, green innovation also considers environmental factors, and taking into account environmental performance is an important difference between green and traditional innovation [49]. Therefore, if an enterprise is engaged in heavily polluting production and operation activities, green innovation’s consideration of environmental performance can enable the enterprise to obtain higher innovation benefits, thereby alleviating the negative impact of green innovation on its green economic efficiency. To verify the above expectations, this paper divides the samples into two groups, “heavily polluting enterprises” and “non-heavily polluting enterprises”, for the regression analysis. Specifically, referring to the practice of Liu et al. (2022) [50], if the overall emissions intensity of the production and operation activities of an enterprise is higher than the critical value, it is classified as a “heavily polluting enterprise”; otherwise, it is classified as a “non-heavily polluting enterprise”. The above two groups of samples are substituted into model (5) for the regression analysis, and the results are shown in columns (3)–(4) of Table 7. The results show that the GI coefficient of the “heavily polluting enterprises” group is −0.0050 and not statistically significant; the coefficient of “non-heavily polluting enterprises” is −0.0316 and statistically significant at the 5% level, indicating that when enterprise engages in heavily polluting production and operation activities, every unit increase in the level of green innovation will statistically insignificantly reduce the green economic efficiency for 0.0050 unit. Otherwise, every unit increase in the level of green innovation will statistically significantly reduce the green economic efficiency by 0.0316 units. This shows that the consideration of environmental performance from green innovation enables heavily polluting enterprises to obtain higher innovation benefits, which in turn alleviates the negative impact of green innovation on their green economic efficiency.

### 4.6. How Green Innovation Can Positively Impact Enterprise Green Economic Efficiency

According to the literature, if the production process cannot be upgraded, the freedom of technology selection is insufficient, the costs are less sensitive or lack of market incentives, innovation compensation will not be realized [13]. In other words, if the Porter hypothesis is established, green innovation should have a significant positive impact on the green economic efficiency of enterprises when the above conditions are met. Accordingly, based on the previous definitions of production process upgrades, degree of freedom of technology selection, and market competition intensity, this paper carries out regression analysis by substituting the combined condition screening samples into model (5). The results are shown in Table 8.

When the enterprise has a high degree of freedom of technology selection and high market competition intensity, and only the production process upgrade cannot be achieved, the result is shown in columns (1) of Table 8. It shows that the coefficient of GI is 0.0151, and the t-value is 1.23, indicating that if the enterprise has a high degree of freedom of technology selection and high market competition intensity, every unit increase in the level of green innovation will statistically insignificantly stimulate the green economic efficiency for 0.0151 unit. This result is better than conditions in which the enterprise only has a high degree of freedom of technology selection (coefficient = −0.0154, t-value = −1.27) or only high market competition intensity (coefficient = −0.0108, t-value = −0.97) because the combination of above two conditions statistically and economically partly makes green innovation stimulating the green economic efficiency.

If the enterprise upgrades its production process, faces high market competition intensity, and has a low degree of freedom of technology selection, the result is shown in columns (2) of Table 8. It shows that the coefficient of GI-I is −0.0091, and the t-value is −0.73, indicating that if an enterprise upgrades its production process and faces high market competition intensity, every unit increase of the level of green innovation will statistically insignificantly reduce the green economic efficiency for 0.0091 unit. This result is better than conditions in which the enterprise only upgrades its production process (coefficient = −0.0096, t-value = −1.45) or only faces high market competition intensity (coefficient = −0.0108, t-value = −0.97), because the combination of the above two conditions statistically and economically further alleviates the adverse effects of green innovation on the green economic efficiency.

Similarly, if the enterprise upgrades its production process, has a high degree of freedom of technology selection, and faces low market competition intensity, the result is shown in columns (3) of Table 8. It shows that the coefficient of GI-I is −0.0086, and the t-value is −0.94, indicating that if an enterprise upgrades its production process and has a high degree of freedom of technology selection, every unit increase in the level of green innovation will statistically insignificantly reduce the green economic efficiency for 0.0086 unit. This result is better than conditions in which the enterprise only upgrades its production process (coefficient = −0.0096, t-value = −1.45) or only has a high degree of freedom of technology selection (coefficient = −0.0154, t-value = −1.27) because the combination of above two conditions statistically and economically further alleviates the adverse effects of green innovation on the green economic efficiency. The two-conditions combination contains production process upgrading can only alleviate the adverse effects of green innovation on green economic efficiency, a possible reason is that production process upgrading increases the economic burden on enterprises [36].

Finally, if the enterprise upgrades its production process, has a high degree of freedom of technology selection, and faces high market competition intensity, the result is shown in columns (4) of Table 8. It shows that the coefficient of GI-I is 0.0196 and is statistically significant at the 10% level, indicating that when all conditions for realizing innovation compensation are met, every unit increase in the level of green innovation will statistically significantly stimulate the green economic efficiency for 0.0196 unit. Consistent with Porter’s hypothesis, the above results show that a combination of conditions for realizing innovation compensation can help reverse or at least alleviate the negative impact of green innovation on the green economic efficiency of enterprises. Specifically, the combination of all conditions will make green innovation have a statistically and economically significant positive impact on green economic efficiency.

## 5. Conclusions, Recommendations, and Limitations

### 5.1. Conclusions

Taking listed companies from 2016 to 2020 as a sample, this paper examined whether and how green innovation can affect their green economic efficiency and the impact of the degree of protection of innovation achievements and the nature of enterprise production and operation activities on their relationship. The study found that green innovation significantly reduces the green economic efficiency of enterprises, and the above conclusions are still valid after the robustness test using propensity score matching, the instrumental variable method, and the Heckman two-stage method. Achieving production process upgrades, a high degree of freedom of technology selection, and high market competition intensity can help alleviate the negative effects of green innovation on the green economic efficiency of enterprises. If the protection of innovation achievements is insufficient, the accumulation of knowledge and technology is insufficient, and the enterprise is not engaged in the production and operation of heavy pollution, the negative effect of green innovation on its green economic efficiency is more significant. When the three conditions of upgraded production processes, a high degree of freedom of technology selection, and high market competition intensity exist at the same time, green innovation significantly promotes the green economic efficiency of enterprises. The above results show that if supporting measures are lacking, the consideration of green innovations of environmental performance will increase the economic burden, limit the optional technologies, and significantly reduces the green economic efficiency of enterprises.

### 5.2. Recommendations for Practice

Based on the research results, this paper puts forward the following recommendations for practice. First, the government should strengthen its support for green innovation, promote the construction of the technology market, increase subsidies for green innovation, improve the preferential policies for green innovation, properly regulate the macroeconomy, and strengthen the construction of the legal system and the protection of innovation achievements. If the production process is upgraded, the freedom of technology selection is improved, and the market competition intensity is increased, the negative impact of green innovation on the green economic efficiency of enterprises is reduced or even reversed to a significant positive effect. The government should improve relevant systems to reduce enterprises’ economic burden and increase their innovation income and enable green innovation to play a role in promoting high-quality development.

Second, when carrying out green innovation, enterprises should focus on upgrading production processes and simultaneously actively carrying out technology accumulation to offset the adverse impact of green innovation on their green economic efficiency. Production process upgrades and the high-level accumulation of knowledge and technology can reduce the negative impact of green innovation on the green economic efficiency of enterprises and are necessary conditions for green innovation to achieve its role in promoting high-quality development. Therefore, enterprises should carry out green innovation while focusing on technology accumulation and production process upgrades to play a positive role in green innovation. Moreover, to mitigate the negative effects of green innovation, enterprises should employ proper production and operation strategy. Specifically, enterprises engaged in heavily polluting production and operation activities should cooperate with policy requirements and actively carry out green innovation. The characteristics of green innovation, taking into account environmental performance, make it immensely beneficial to heavily polluting enterprises. Heavily polluting enterprises should take the initiative to assume social responsibilities and contribute to the realization of high-quality development.

**Figure 2:**
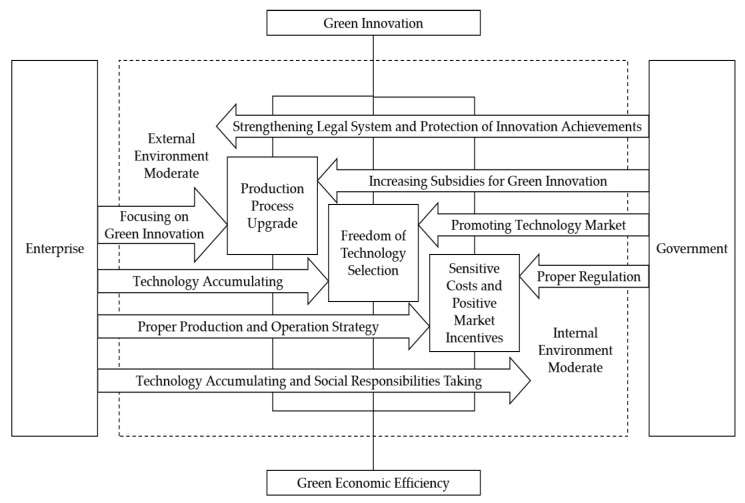
Effects of government support and enterprise strategy.

Based on the above discussions, a graph to trace the effects of government support and enterprise strategy is developed, which is shown in Figure 2.

### 5.3. Limitations

There are some limitations in this paper, which may become indications for future research. First, not every enterprise is mandated to disclose information on pollutant discharges. Constraints on data availability may influence the robustness of this paper’s conclusions. Second, due to some enterprises only disclose the emissions of their main pollutants, such as sulphur dioxide, nitrogen oxide, and chemical oxygen demand, ammonia nitrogen. This paper can only estimate the green economic efficiency of enterprises by using main pollutants instead of all pollutants. The above problem may influence the reliability of estimations results.

## Figures and Tables

**Table 1:** Descriptive statistics.

Variables	Obs	Mean	SD	Median	Min	Max
GEE	2163	0.0840	0.0928	0.0546	0.0007	0.6145
GI	2163	0.1238	0.1696	0.0606	0.0000	0.8462
Size	2163	22.5569	1.2759	22.3884	20.1819	25.8538
ROA	2163	0.0520	0.0502	0.0398	−0.2719	0.2497
Lev	2163	0.3623	0.1931	0.3455	0.0150	0.9584
APTR	2163	10.7435	19.1876	5.8323	0.5973	156.3812
Growth	2163	0.1371	0.2295	0.0816	−0.3311	1.4300
PER	2163	51.2148	75.5002	27.8139	4.0473	512.7808
MF	2163	0.1186	0.4042	0.0532	0.0059	5.7002
RT	2163	0.0179	0.0288	0.0094	0.0001	0.2694
Share	2163	0.3582	0.1495	0.3379	0.0998	0.7505
Inde	2163	0.3711	0.0519	0.3333	0.3333	0.5714
CEO	2163	0.2367	0.4252	0	0	1
Age	2163	2.9961	0.2640	3.0445	2.1972	3.4657

**Table 2:** Green innovation and green economic efficiency.

Variables	No Control	Financial Controls	Governance Controls	All Controls
	(1)	(2)	(3)	(4)
	GEE	GEE	GEE	GEE
GI	−0.0176 **	−0.0159 **	−0.0177 **	−0.0161 **
	(−2.26)	(−2.12)	(−2.30)	(−2.16)
Financial Controls		Yes		Yes
Governance Controls			Yes	Yes
Year fixed effect	Yes	Yes	Yes	Yes
Firm fixed effect	Yes	Yes	Yes	Yes
N	2163	2163	2163	2163
Adj_R^2^	0.0265	0.0745	0.0295	0.0774

Note: ** represent significance at the 5% level, and the t-values are in brackets.

**Table 3:** Green innovation and green economic efficiency: PSM.

Variables	1:1 Match		1:4 Match	
	(1)	(2)	(3)	(4)
	GEE	GEE	GEE	GEE
GI	−0.0289 ***	−0.0312 ***	−0.0177 **	−0.0178 **
	(−2.87)	(−2.98)	(−2.26)	(−2.25)
Controls		Yes		Yes
Year fixed effect	Yes	Yes	Yes	Yes
Firm fixed effect	Yes	Yes	Yes	Yes
N	1238	1238	1872	1872
Adj_R^2^	0.0410	0.0833	0.0329	0.0653

Note: ** and *** represent significance at the 5% and 1% levels, respectively, and the t-values are in brackets.

**Table 4:** Green innovation and green economic efficiency: 2SLS and Heckman two-stage.

Variables	2SLS		Heckman Two-Stage	
	First Stage	Second Stage	First Stage	Second Stage
	(1)	(2)	(3)	(4)
	GI	GEE	GI	GEE
IV	0.9797 ***		8.6956 ***	
	(34.39)		(18.62)	
GI		−0.0163 *		−0.0213 **
		(−1.66)		(−2.54)
IMR				0.0025 *
				(1.69)
Controls	Yes	Yes	Yes	Yes
Year fixed effect	Yes	Yes	Yes	Yes
Firm fixed effect	Yes	Yes	Yes	Yes
N	2091	2091	2225	2163
Pseudo/Adj_R^2^	0.5559	0.0774	0.2872	0.0786
Kleibergen–Paap rk LM Statistic	59.759			
Cragg-Donald Wald F Statistic	1762.702			
Kleibergen–Paap rk Wald F Statistic	1182.870			

Note: *, **, and *** represent significance at the 10%, 5%, and 1% levels, respectively, and the t-values are in brackets.

**Table 5:** Influence of innovation form, freedom of technological selection, and market competition intensity.

Variables	Green Invention Innovation	Green Utility Model Innovation	High-Tech Market	Low-Tech Market	High Market Competition	Low Market Competition
	(1)	(2)	(3)	(4)	(5)	(6)
	GEE	GEE	GEE	GEE	GEE	GEE
GI-I	−0.0096					
	(−1.45)					
GI-UM		−0.0142 **				
		(−2.35)				
GI			−0.0154	−0.0199 **	−0.0108	−0.0185 *
			(−1.27)	(−2.00)	(−0.97)	(−1.92)
Controls	Yes	Yes	Yes	Yes	Yes	Yes
Year fixed effect	Yes	Yes	Yes	Yes	Yes	Yes
Firm fixed effect	Yes	Yes	Yes	Yes	Yes	Yes
N	2108	2108	862	1301	1191	972
Adj_R^2^	0.0638	0.0653	0.1373	0.0961	0.1256	0.0633

Note: * and ** represent significance at the 10% and 5% levels, respectively, and the t-values are in brackets.

**Table 6:** Influence of the degree of protection of innovation achievements.

Variables	Strong Legal Environment	Weak Legal Environment	Strong Technical Protection	Weak Technical Protection
	(1)	(2)	(3)	(4)
	GEE	GEE	GEE	GEE
GI	−0.0067	−0.0152 *	−0.0129	−0.0174 *
	(−0.50)	(−1.90)	(−1.23)	(−1.71)
Controls	Yes	Yes	Yes	Yes
Year fixed effect	Yes	Yes	Yes	Yes
Firm fixed effect	Yes	Yes	Yes	Yes
N	933	1230	987	1176
Adj_R^2^	0.0933	0.1494	0.0803	0.1255

Note: * represent significance at the 10% level, and the t-values are in brackets.

**Table 7:** Influence of the nature of production and operation activities of enterprises.

Variables	High-Tech Enterprise	Nonhigh-Tech Enterprise	Heavily Polluting Enterprise	Nonheavily Polluting Enterprise
	(1)	(2)	(3)	(4)
	GEE	GEE	GEE	GEE
GI	−0.0108	−0.0171 **	−0.0050	−0.0316 **
	(−0.73)	(−2.10)	(−0.65)	(−2.33)
Controls	Yes	Yes	Yes	Yes
Year fixed effect	Yes	Yes	Yes	Yes
Firm fixed effect	Yes	Yes	Yes	Yes
N	1026	1137	749	1414
Adj_R^2^	0.1089	0.0929	0.1926	0.0762

Note: ** represent significance at the 5% level, and the t-values are in brackets.

**Table 8:** The positive impact of green innovation on green economic efficiency.

Variables	Lack of Production Process Upgrade	Lack of High Degree of Freedom of Technology Selection	Lack of High Market Competition Intensity	All Conditions Are Met
	(1)	(2)	(3)	(4)
	GEE	GEE	GEE	GEE
GI	0.0151			
	(1.23)			
GI-I		−0.0091	−0.0086	0.0196 *
		(−0.73)	(−0.94)	(1.95)
Controls	Yes	Yes	Yes	Yes
Year fixed effect	Yes	Yes	Yes	Yes
Firm fixed effect	Yes	Yes	Yes	Yes
N	489	1191	862	489
Adj_R^2^	0.3316	0.1255	0.1366	0.3333

Note: * represent significance at the 10% level, and the t-values are in brackets.

## Data Availability

The data used in this research are available on the following websites: https://www.gtarsc.com/ (accessed on 25 August 2022); https://www.cnrds.com/ (accessed on 30 August 2022); http://www.cninfo.com.cn (accessed on 10 August 2022).

## Appendix A

The results of statistical analysis are sensitive to outliers data. In order to exclude the interference of outliers data. For all continuous variables, this paper replaces the maximum 1% data with 99% percentile and replaces the minimum 1% data with 1% percentile.

## Appendix B

**Table A1:** Variable definitions.

Variable Symbol	Variable Name	Variable Definitions
GEE	Green economic efficiency	See definition above
GI	Green innovation	Number of green patent applications/total number of patent applications
Size	Enterprise size	Natural logarithm of total assets of the enterprise
ROA	Return on assets	Net profit/Total assets
Lev	Asset-liability ratio	Total liabilities/Total assets
APTR	Accounts payable turnover ratio	Operating costs/Accounts payable
Growth	Growth	(End value of the total assets-Beginning value of total assets)/(Beginning value of total assets)
PER	Price-earnings ratio	Closing price today/(Net profit in previous year’s annual report/Value of paid-in capital at end of the current period)
MF	Management fees rate	Management costs/Operating revenue
RT	Risk-taking level	The standard deviation of return on assets of enterprise in periods t − 1, t, and t + 1
Share	Ownership concentration	Number of shares held by largest shareholder/Number of shares held by all shareholders
Inde	The proportion of independent directors	Number of independent directors/Total number of board of directors
CEO	Two-in-one	Equals 1 if the chairman of the board is also the general manager; otherwise, equals 0
Age	Enterprise age	Natural logarithm of years since the company’s establishment

**Table A2:** Descriptive statistics of input and output.

Variables	Obs	Mean	SD	Median	Min	Max
Capital Input	2163	2.21 × 10^9^	5.53 × 10^8^	5.85 × 10^9^	223,369	4.40 × 10^10^
Labor Input	2163	10,331	3813	24,715	153	508,757
Desired Output	2163	6.21 × 10^9^	1.89 × 10^9^	1.28 × 10^10^	5,363,377	7.99 × 10^10^
Undesired Output	2163	2,778,327	138,168	10,002,373	88	74,455,192

**Table A3:** Green innovation and green economic efficiency (Full version of Table 2).

Variables	No Control	Financial Controls	Governance Controls	All Controls
	(1)	(2)	(3)	(4)
	GEE	GEE	GEE	GEE
GI	−0.0176 **	−0.0159 **	−0.0177 **	−0.0161 **
	(−2.26)	(−2.12)	(−2.30)	(−2.16)
Size		−0.0220		−0.0208 *
		(−1.62)		(−1.66)
ROA		0.0862 **		0.0892 **
		(2.22)		(2.37)
Lev		−0.0024		−0.0024
		(−0.15)		(−0.14)
APTR		0.0004		0.0004
		(1.18)		(1.22)
Growth		0.0114 *		0.0105 *
		(1.67)		(1.68)
PER		−0.0000		−0.0000
		(−1.35)		(−1.38)
MF		−0.0203 **		−0.0203 **
		(−2.38)		(−2.42)
RT		−0.0793 *		−0.0749*
		(−1.88)		(−1.80)
Share			−0.0446	−0.0580
			(−0.71)	(−1.02)
Inde			0.0151	−0.0036
			(0.36)	(−0.09)
CEO			−0.0014	−0.0011
			(−0.32)	(−0.26)
Age			−0.0783	−0.0503
			(−1.18)	(−0.86)
_cons	0.0682 ***	0.5581 *	0.3040	0.6981 *
	(19.57)	(1.82)	(1.51)	(1.78)
Year fixed effect	Yes	Yes	Yes	Yes
Firm fixed effect	Yes	Yes	Yes	Yes
N	2163	2163	2163	2163
Adj_R^2^	0.0265	0.0745	0.0295	0.0774

Note: *, **, and *** represent significance at the 10%, 5%, and 1% levels, respectively, and the t-values are in brackets.

**Table A4:** Balance hypothesis test: 1:1.

Variables	Match	Mean		Bias	Reduct Bias	t-Value	*p*-Value
		Treated	Control				
Size	Unmatched	22.877	22.390	37.9	78.8	8.59	0.000
	Matched	22.877	22.774	8.0		1.51	0.132
ROA	Unmatched	0.049	0.054	−10.4	93.2	−2.30	0.021
	Matched	0.049	0.048	0.7		0.14	0.887
Lev	Unmatched	0.388	0.351	19.0	51.6	4.24	0.000
	Matched	0.388	0.370	9.2		1.76	0.078
APTR	Unmatched	12.803	9.657	15.4	42.8	3.66	0.000
	Matched	12.803	14.601	−8.8		−1.30	0.193
Growth	Unmatched	0.134	0.140	−2.5	−164.4	−0.55	0.585
	Matched	0.134	0.149	−6.6		−1.30	0.193
PER	Unmatched	45.877	54.370	−11.4	86.3	−2.48	0.013
	Matched	45.877	44.716	1.6		0.34	0.734
MF	Unmatched	0.130	0.112	4.3	−687.8	0.97	0.332
	Matched	0.130	0.268	−34.0		−3.55	0.000
TobinQ	Unmatched	1.572	1.886	−29.1	88.1	−6.24	0.000
	Matched	1.572	1.609	−3.5		−0.76	0.445
Share	Unmatched	0.358	0.359	−0.5	−597.6	−0.12	0.905
	Matched	0.358	0.352	3.7		0.73	0.464
Inde	Unmatched	0.370	0.372	−4.8	−47.7	−1.07	0.283
	Matched	0.370	0.373	−7.1		−1.35	0.177
CEO	Unmatched	0.210	0.251	−9.6	17.1	−2.11	0.035
	Matched	0.210	0.244	−8.0		−1.54	0.123
Age	Unmatched	2.971	3.011	−14.8	74.1	−3.33	0.001
	Matched	2.971	2.961	3.8		0.73	0.465

**Table A5:** Balance hypothesis test: 1:4.

Variables	Match	Mean		Bias	Reduct Bias	t-Value	*p*-Value
		Treated	Control				
Size	Unmatched	22.877	22.390	37.9	85.2	8.59	0.000
	Matched	22.877	22.805	5.6		1.04	0.298
ROA	Unmatched	0.049	0.054	−10.4	98.1	−2.30	0.021
	Matched	0.049	0.048	0.2		0.04	0.968
Lev	Unmatched	0.388	0.351	19.0	74.7	4.24	0.000
	Matched	0.388	0.378	4.8		0.91	0.361
APTR	Unmatched	12.803	9.657	15.4	76.4	3.66	0.000
	Matched	12.803	13.547	−3.6		−0.57	0.568
Growth	Unmatched	0.134	0.140	−2.5	−50.0	−0.55	0.585
	Matched	0.134	0.143	−3.7		−0.72	0.473
PER	Unmatched	45.877	54.370	−11.4	67.7	−2.48	0.013
	Matched	45.877	48.622	−3.7		−0.74	0.460
MF	Unmatched	0.130	0.112	4.3	−543.0	0.97	0.332
	Matched	0.130	0.243	−27.8		−3.19	0.001
TobinQ	Unmatched	1.572	1.886	−29.1	89.6	−6.24	0.000
	Matched	1.572	1.604	−3.0		−0.68	0.497
Share	Unmatched	0.358	0.359	−0.5	−296.9	−0.12	0.905
	Matched	0.358	0.355	2.1		0.41	0.680
Inde	Unmatched	0.370	0.372	−4.8	8.8	−1.07	0.283
	Matched	0.370	0.367	4.4		0.86	0.388
CEO	Unmatched	0.210	0.251	−9.6	86.7	−2.11	0.035
	Matched	0.210	0.216	−1.3		−0.25	0.801
Age	Unmatched	2.971	3.011	−14.8	82.9	−3.33	0.001
	Matched	2.971	2.978	−2.5		−0.48	0.630

**Table A6:** Average treatment effect on the treated: 1:1.

Variables	Sample	Treated	Controls	Difference	S.E.	T-Stat
GEE	Unmatched	0.083	0.085	−0.002	0.004	−0.55
	ATT	0.083	0.098	−0.015	0.007	−2.34

**Table A7:** Average treatment effect on the treated: 1:4.

Variables	Sample	Treated	Controls	Difference	S.E.	T-Stat
GEE	Unmatched	0.083	0.085	−0.002	0.004	−0.55
	ATT	0.083	0.095	−0.012	0.005	−2.34

**Table A8:** Green innovation and green economic efficiency: PSM (Full version of Table 3).

Variables	1:1 Match		1:4 Match	
	(1)	(2)	(3)	(4)
	GEE	GEE	GEE	GEE
GI	−0.0289 ***	−0.0312 ***	−0.0177 **	−0.0178 **
	(−2.87)	(−2.98)	(−2.26)	(−2.25)
Size		−0.0117		−0.0126
		(−0.69)		(−0.83)
ROA		0.0474		0.0977 **
		(0.78)		(2.31)
Lev		−0.0287		0.0006
		(−1.00)		(0.04)
APTR		0.0003		0.0003
		(0.96)		(1.07)
Growth		0.0076		0.0059
		(1.12)		(1.21)
PER		−0.0000		−0.0000
		(−0.59)		(−0.41)
MF		−0.0079		−0.0109 ***
		(−1.59)		(−3.13)
RT		−0.0966 *		−0.1183 ***
		(−1.69)		(−2.60)
Share		−0.0818		−0.0636
		(−1.15)		(−1.02)
Inde		0.0295		0.0043
		(0.63)		(0.10)
CEO		−0.0106		−0.0020
		(−1.57)		(−0.45)
Age		−0.0991		−0.0886
		(−1.13)		(−1.36)
_cons	0.0726 ***	0.6460	0.0686 ***	0.6206
	(14.84)	(1.14)	(17.95)	(1.33)
Year fixed effect	Yes	Yes	Yes	Yes
Firm fixed effect	Yes	Yes	Yes	Yes
N	1238	1238	1872	1872
Adj_R^2^	0.0410	0.0833	0.0329	0.0653

Note: *, **, and *** represent significance at the 10%, 5%, and 1% levels, respectively, and the t-values are in brackets.

**Table A9:** Correlation coefficients of IV and GEE.

Variables	GEE	IV
GEE	1.000	
IV	0.025	1.000
	(0.242)	

Note: the *p*-values are in brackets.

**Table A10:** Green innovation and green economic efficiency: 2SLS (Full version of column (1)–(2) of Table 4).

Variables	First Stage	Second Stage
	(1)	(2)
	GI	GEE
IV	0.9797 ***	
	(34.39)	
GI		−0.0163 *
		(−1.66)
Size	−0.0190	−0.0208 *
	(−0.82)	(−1.67)
ROA	0.0602	0.0892 **
	(0.69)	(2.37)
Lev	0.0679 *	−0.0024
	(1.87)	(−0.15)
APTR	0.0001	0.0004
	(0.70)	(1.22)
Growth	−0.0025	0.0105 *
	(−0.21)	(1.68)
PER	0.0000	−0.0000
	(0.11)	(−1.38)
MF	0.0317 *	−0.0203 **
	(1.90)	(−2.46)
RT	0.0796	−0.0748 *
	(0.70)	(−1.79)
Share	0.0054	−0.0580
	(−0.12)	(−1.02)
Inde	−0.0348	−0.0036
	(−0.42)	(−0.09)
CEO	−0.0165	−0.0011
	(−1.52)	(−0.26)
Age	0.0908	−0.0503
	(0.63)	(−0.86)
Year fixed effect	Yes	Yes
Firm fixed effect	Yes	Yes
N	2091	2091
Adj_R^2^	0.5559	0.0774
Kleibergen–Paap rk LM Statistic	59.759	
Cragg-Donald Wald F Statistic	1762.702	
Kleibergen–Paap rk Wald F Statistic	1182.870	

Note: *, **, and *** represent significance at the 10%, 5%, and 1% levels, respectively, and the t-values are in brackets.

**Table A11:** Green innovation and green economic efficiency: Heckman two-stage (Full version of column (3)–(4) of Table 4).

Variables	First Stage	Second Stage
	(1)	(2)
	GI	GEE
IV	8.6956 ***	
	(18.62)	
GI		−0.0213 **
		(−2.54)
IMR		0.0025 *
		(1.69)
Size	0.4133 ***	−0.0208 *
	(12.37)	(−1.66)
ROA	0.6644	0.0899 **
	(0.95)	(2.38)
Lev	0.1491	−0.0028
	(0.76)	(−0.17)
APTR	−0.0012	0.0004
	(−0.68)	(1.20)
Growth	−0.0638	0.0105 *
	(−0.48)	(1.67)
PER	−0.0006	−0.0000
	(−1.45)	(−1.41)
Share	−0.3249	−0.0573
	(−1.43)	(−1.01)
Inde	0.2586	−0.0045
	(0.42)	(−0.12)
Age	−0.1253	−0.0500
	(−0.98)	(−0.85)
MF		−0.0206 **
		(−2.42)
RT		−0.0754 *
		(−1.81)
CEO		−0.0011
		(−0.25)
_cons	−9.3237 ***	0.6978 *
	(−11.73)	(1.77)
Year fixed effect	Yes	Yes
Firm fixed effect	Yes	Yes
N	2225	2163
Pseudo/Adj_R^2^	0.2872	0.0786

Note: *, **, and *** represent significance at the 10%, 5%, and 1% levels, respectively, and the t-values are in brackets.

**Table A12:** Influence of innovation form, freedom of technological selection, and market competition intensity (Full version of Table 5).

Variables	Green Invention Innovation	Green Utility Model Innovation	High-Tech Market	Low-Tech Market	High Market Competition	Low Market Competition
	(1)	(2)	(3)	(4)	(5)	(6)
	GEE	GEE	GEE	GEE	GEE	GEE
GI-I	−0.0096					
	(−1.45)					
GI-UM		−0.0142 **				
		(−2.35)				
GI			−0.0154	−0.0199 **	−0.0108	−0.0185 *
			(−1.27)	(−2.00)	(−0.97)	(−1.92)
Size	−0.0224 *	−0.0225 *	−0.0118	−0.0234	−0.0408 *	0.0032
	(−1.70)	(−1.71)	(−0.88)	(−1.32)	(−1.82)	(0.22)
ROA	0.0956 **	0.0964 **	0.0680	0.0620	0.0755	0.1248 **
	(2.40)	(2.44)	(1.23)	(1.13)	(1.42)	(2.51)
Lev	−0.0016	−0.0006	0.0069	−0.0087	−0.0005	0.0064
	(−0.10)	(−0.04)	(0.20)	(−0.42)	(−0.03)	(0.25)
APTR	0.0004	0.0004	0.0000	0.0007	0.0005	0.0002
	(1.17)	(1.21)	(0.02)	(1.15)	(0.95)	(1.50)
Growth	0.0125 *	0.0127 *	−0.0008	0.0157	0.0182 **	−0.0025
	(1.72)	(1.75)	(−0.09)	(1.52)	(2.14)	(−0.31)
PER	−0.0000	−0.0000 *	−0.0001 **	0.0000	−0.0001 *	0.0000
	(−1.64)	(−1.67)	(−2.52)	(0.13)	(−1.86)	(0.53)
MF	−0.0128 ***	−0.0117 ***	−0.0381 *	−0.0121 **	−0.0314 **	−0.0095 ***
	(−2.73)	(−2.67)	(−1.86)	(−2.29)	(−2.21)	(−3.08)
RT	−0.0741 *	−0.0720 *	0.0966	−0.1432 **	−0.0645	−0.1199 **
	(−1.78)	(−1.69)	(1.13)	(−2.36)	(−1.10)	(−2.17)
Share	−0.0597	−0.0633	−0.0855	−0.0525	−0.0793	−0.0685
	(−1.04)	(−1.10)	(−1.20)	(−0.53)	(−0.77)	(−1.09)
Inde	−0.0055	−0.0058	−0.0525	0.0113	0.0106	−0.0085
	(−0.14)	(−0.15)	(−0.61)	(0.25)	(0.22)	(−0.13)
CEO	−0.0002	0.0001	0.0096	−0.0100 **	0.0049	−0.0064
	(−0.03)	(0.01)	(1.57)	(−2.02)	(0.75)	(−1.00)
Age	−0.0455	−0.0382	−0.2079 *	−0.0658	−0.0849	0.0080
	(−0.68)	(−0.58)	(−1.94)	(−0.76)	(−0.88)	(0.14)
_cons	0.7211 *	0.7021 *	0.9899 **	0.7855	1.2360 *	−0.0068
	(1.73)	(1.69)	(2.32)	(1.30)	(1.86)	(−0.02)
Year fixed effect	Yes	Yes	Yes	Yes	Yes	Yes
Firm fixed effect	Yes	Yes	Yes	Yes	Yes	Yes
N	2108	2108	862	1301	1191	972
Adj_R^2^	0.0638	0.0653	0.1373	0.0961	0.1256	0.0633

Note: *, **, and *** represent significance at the 10%, 5%, and 1% levels, respectively, and the t-values are in brackets.

**Table A13:** Influence of the degree of protection of innovation achievements (Full version of Table 6).

Variables	Strong Legal Environment	Weak Legal Environment	Strong Technical Protection	Weak Technical Protection
	(1)	(2)	(3)	(4)
	GEE	GEE	GEE	GEE
GI	−0.0067	−0.0152 *	−0.0129	−0.0174 *
	(−0.50)	(−1.90)	(−1.23)	(−1.71)
Size	0.0129	−0.0092	−0.0081	−0.0148
	(0.90)	(−0.84)	(−0.56)	(−1.28)
ROA	0.0787	0.0404	0.0221	0.1189 ***
	(1.27)	(0.98)	(0.40)	(3.00)
Lev	−0.0108	−0.0079	−0.0219	0.0112
	(−0.40)	(−0.40)	(−0.77)	(0.52)
APTR	0.0006	0.0002	0.0007	0.0001
	(1.08)	(1.36)	(1.14)	(0.80)
Growth	0.0035	0.0085	0.0189	0.0100 **
	(0.43)	(1.60)	(1.28)	(2.06)
PER	−0.0000	−0.0000 *	−0.0000	−0.0001
	(−0.88)	(−1.92)	(−1.12)	(−1.31)
MF	−0.0039	−0.0396 *	−0.0078 *	−0.0275 **
	(−0.46)	(−1.79)	(−1.89)	(−2.42)
RT	−0.0255	−0.0788	−0.0346	−0.0601
	(−0.25)	(−1.37)	(−0.45)	(−1.15)
Share	0.0273	0.0417	−0.0617	−0.0459
	(0.40)	(1.00)	(−0.53)	(−0.91)
Inde	0.0435	−0.0751	0.0448	−0.0635
	(0.70)	(−1.57)	(0.86)	(−1.40)
CEO	−0.0025	−0.0052	−0.0091	0.0045
	(−0.60)	(−0.94)	(−1.35)	(0.87)
Age	−0.2055 **	0.0309	−0.0948	0.0450
	(−1.97)	(0.51)	(−1.19)	(0.80)
_cons	0.3453	0.1998	0.5283	0.3069
	(0.91)	(0.66)	(1.17)	(1.09)
Year fixed effect	Yes	Yes	Yes	Yes
Firm fixed effect	Yes	Yes	Yes	Yes
N	933	1230	987	1176
Adj_R^2^	0.0933	0.1494	0.0803	0.1255

Note: *, **, and *** represent significance at the 10%, 5%, and 1% levels, respectively, and the t-values are in brackets.

**Table A14:** Influence of the nature of production and operation activities of enterprises (Full version of Table 7).

Variables	High-Tech Enterprise	Nonhigh-Tech Enterprise	Heavily Polluting Enterprise	Nonheavily Polluting Enterprise
	(1)	(2)	(3)	(4)
	GEE	GEE	GEE	GEE
GI	−0.0108	−0.0171 **	−0.0050	−0.0316 **
	(−0.73)	(−2.10)	(−0.65)	(−2.33)
Size	−0.0463 *	−0.0006	−0.0394	0.0019
	(−1.80)	(−0.05)	(−1.57)	(0.22)
ROA	0.0430	0.1675 ***	0.0512	0.0885 **
	(0.79)	(4.10)	(0.68)	(2.16)
Lev	0.0125	−0.0090	−0.0612 *	0.0169
	(0.54)	(−0.44)	(−1.90)	(0.83)
APTR	0.0005	0.0002 *	0.0000	0.0006
	(0.87)	(1.92)	(0.01)	(1.32)
Growth	0.0175 *	0.0014	0.0142	0.0072
	(1.93)	(0.22)	(1.05)	(1.25)
PER	−0.0001	−0.0000	−0.0001	−0.0000
	(−1.39)	(−0.64)	(−1.16)	(−0.86)
MF	−0.0332 **	−0.0093 ***	−0.0426 ***	−0.0053
	(−2.27)	(−3.47)	(−2.95)	(−1.57)
RT	−0.0911	−0.1013 *	−0.0651	−0.0777 *
	(−1.33)	(−1.82)	(−0.59)	(−1.77)
Share	−0.0775	−0.0569	−0.1376	0.0596
	(−0.55)	(−1.15)	(−1.64)	(0.95)
Inde	−0.0365	0.0239	0.0646	−0.0176
	(−0.48)	(0.58)	(0.96)	(−0.37)
CEO	0.0099	−0.0102 *	0.0093	−0.0061
	(1.40)	(−1.72)	(1.08)	(−1.21)
Age	−0.1001	−0.0044	−0.1153	−0.0047
	(−0.95)	(−0.08)	(−0.87)	(−0.08)
_cons	1.3980 *	0.1098	1.3236	0.0161
	(1.82)	(0.37)	(1.64)	(0.07)
Year fixed effect	Yes	Yes	Yes	Yes
Firm fixed effect	Yes	Yes	Yes	Yes
N	1026	1137	749	1414
Adj_R^2^	0.1089	0.0929	0.1926	0.0762

Note: *, **, and *** represent significance at the 10%, 5%, and 1% levels, respectively, and the t-values are in brackets.

**Table A15:** The positive impact of green innovation on green economic efficiency (Full version of Table 8).

Variables	Lack of Production Process Upgrade	Lack of High Degree of Freedom of Technology Selection	Lack of High Market Competition Intensity	All Conditions Are Met
	(1)	(2)	(3)	(4)
	GEE	GEE	GEE	GEE
GI	0.0151			
	(1.23)			
GI-I		−0.0091	−0.0086	0.0196 *
		(−0.73)	(−0.94)	(1.95)
Size	−0.0308	−0.0408 *	−0.0121	−0.0312
	(−1.53)	(−1.83)	(−0.92)	(−1.56)
ROA	0.0788	0.0745	0.0690	0.0780
	(1.56)	(1.39)	(1.26)	(1.49)
Lev	0.0006	−0.0009	0.0061	0.0018
	(0.02)	(−0.05)	(0.18)	(0.06)
APTR	−0.0002	0.0005	−0.0000	−0.0002
	(−1.38)	(0.94)	(−0.09)	(−1.28)
Growth	0.0064	0.0183 **	−0.0003	0.0059
	(0.72)	(2.16)	(−0.04)	(0.67)
PER	−0.0001 **	−0.0001 *	−0.0001 **	−0.0001 **
	(−2.00)	(−1.85)	(−2.51)	(−2.04)
MF	−0.0815 ***	−0.0319 **	−0.0381 *	−0.0815 ***
	(−4.27)	(−2.29)	(−1.86)	(−4.28)
RT	0.1007	−0.0648	0.0938	0.1005
	(1.06)	(−1.12)	(1.11)	(1.05)
Share	0.0064	−0.0786	−0.0836	0.0023
	(0.11)	(−0.76)	(−1.19)	(0.04)
Inde	0.0534	0.0114	−0.0511	0.0557
	(0.62)	(0.24)	(−0.60)	(0.65)
CEO	0.0058	0.0049	0.0095	0.0063
	(0.91)	(0.76)	(1.54)	(0.99)
Age	−0.1338	−0.0875	−0.2045 *	−0.1303
	(−0.95)	(−0.89)	(−1.92)	(−0.93)
_cons	1.1184 *	1.2422 *	0.9861 **	1.1177 *
	(1.74)	(1.87)	(2.32)	(1.74)
Year fixed effect	Yes	Yes	Yes	Yes
Firm fixed effect	Yes	Yes	Yes	Yes
N	489	1191	862	489
Adj_R^2^	0.3316	0.1255	0.1366	0.3333

Note: *, **, and *** represent significance at the 10%, 5%, and 1% levels, respectively, and the t-values are in brackets.
